# Radiation-modulated thermoelectric fabrics for wearable energy harvesting

**DOI:** 10.1093/nsr/nwaf215

**Published:** 2025-05-31

**Authors:** Yalong Wang, Hu Liu, Shiliang Zhang, Guangming Tao, Chuntai Liu, Changyu Shen

**Affiliations:** State Key Laboratory of Structural Analysis, Optimization and CAE Software for Industrial Equipment; National Engineering Research Center for Advanced Polymer Processing Technology, Zhengzhou University, China; State Key Laboratory of Structural Analysis, Optimization and CAE Software for Industrial Equipment; National Engineering Research Center for Advanced Polymer Processing Technology, Zhengzhou University, China; State Key Laboratory of New Textile Materials and Advanced Processing & Research Center for Intelligent Fiber Devices and Equipment, School of Medical Equipment Science and Engineering, Huazhong University of Science and Technology, China; State Key Laboratory of New Textile Materials and Advanced Processing & Research Center for Intelligent Fiber Devices and Equipment, School of Medical Equipment Science and Engineering, Huazhong University of Science and Technology, China; Department of Geriatrics and Key Laboratory of Vascular Aging, Ministry of Education, Tongji Hospital of Tongji Medical College, Huazhong University of Science and Technology, China; State Key Laboratory of Structural Analysis, Optimization and CAE Software for Industrial Equipment; National Engineering Research Center for Advanced Polymer Processing Technology, Zhengzhou University, China; State Key Laboratory of Structural Analysis, Optimization and CAE Software for Industrial Equipment; National Engineering Research Center for Advanced Polymer Processing Technology, Zhengzhou University, China

Flexible wearable thermoelectric generator (TEG) fabrics can convert body heat or solar energy into sustainable electricity, advancing the development of self-powered wearable electronics. To achieve high energy-conversion efficiency, strategies such as band convergence, alloy solid solutions and low-dimensional material engineering [1–3] have been employed to optimize the intrinsic Seebeck coefficient and thermoelectric figure of merit (*ZT*). However, challenges persist in manufacturing on a commercial scale and establishing high temperature gradients (*ΔT*).

With rapid advancements in photothermal-heating [4,5] and radiative-cooling technologies [6,7], a radiation modulation strategy for TEG has been developed to create large *ΔT* by leveraging solar energy. Thermoelectric materials with photothermal properties significantly increase the hot-side temperature of thermoelectric legs, while the radiation cooling film with high reflectivity and atmospheric transparent window emissivity lower the cold-side temperature of thermoelectric legs covered with radiative-cooling membranes. By combining photothermal-heating and radiative-cooling technology, the radiation-modulated TEG can produce large *ΔT* under solar radiation and realize efficient solar power generation. Additionally, screen-printing enables the high-precision, patterned and large-scale deposition of thermoelectric pastes onto flexible substrates, providing a simple and scalable solution for low-cost, large-area thermoelectric array fabrication [8,9]. Researchers Liang-Sheng Liao, Ming-Peng Zhuo and Ke-Qin Zhang from Soochow University proposed a simple method for the large-area fabrication of flexible TEG fabrics, achieving high *ΔT* and efficient solar energy conversion [10].

**Figure 1.**
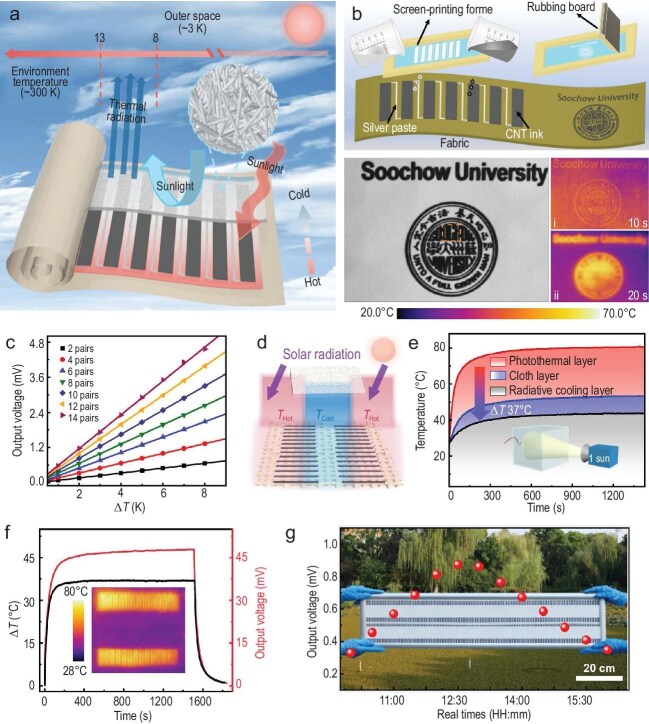
(a) Structure and working principle of wearable TEG fabrics. (b) Schematic of the screen-printing process and infrared images of the university logo under varying illumination times. (c) Output voltage variation with thermoelectric array pairs and *ΔT*. (d) TEG fabric array and radiation-modulated fabric schematic. (e) Temperatures of the photothermal layer, cloth layer and radiative-cooling layer under 1-sun illumination (ambient temperature: 25°C). (f) *ΔT* and output voltage of TEG fabric with a 28-pair thermoelectric array under 1-sun simulated sunlight intensity. (g) Real-time output voltage data of the TEG fabric under natural sunlight in Suzhou, China (7 September 2023). Adapted with permission from ref. [10].

As illustrated in Fig. 1a, under sunlight, the carbon nanotube (CNT) photothermal film in the TEG fabric significantly raises the hot-side temperature due to its photothermal properties, while the poly(vinylidene fluoride-co-hexafluoropropylene) (PVDF-HFP) nanofiber membrane lowers the cold-side temperature via radiative cooling, generating a substantial *ΔT* for efficient solar power generation. Figure 1b shows the process of screen-printing CNT thermoelectric arrays and the ‘Soochow University’ logo on commercial white fabric, which demonstrates the excellent flexibility and high resolution of screen-printing technology. The Soochow University logo on commercial white fabric reaches 70°C after 20 seconds of simulated sunlight exposure, confirming the high photothermal conversion efficiency of screen-printed CNTs. As shown in Fig. 1c, the printed CNT arrays exhibit outstanding thermoelectric performance—when *ΔT* increases from 1 to 8 K in a 14-pair CNT thermoelectric array, the output voltage rises sharply from 0.58 to 4.63 mV. Furthermore, the fabricated thermoelectric fabric exhibits exceptional laundering durability and maintains robust performance under sustained mechanical strain, making it highly suitable for practical smart wearable applications. As shown in the schematic diagram of Fig. 1d, by covering the cold end of the CNT thermoelectric legs with PVDF-HFP nanofiber membrane, a radiative-cooling zone is formed under sunlight, creating a substantial *ΔT* relative to the sunlight-irradiated hot zone of the CNT thermoelectric legs. Under 1-sun air mass (AM) 1.5 illumination, the *ΔT* between the CNT photothermal layer and the radiative-cooling layer reaches 37°C (Fig. 1e), underscoring the synergistic potential of combining photothermal- and radiative-cooling effects. This radiation-modulated TEG fabric leverages the complementary roles of photothermal CNT arrays and radiative-cooling PVDF-HFP membranes to achieve effective personal thermal management, offering enhanced thermal comfort for human users.

A 10 cm × 10 cm radiation-modulated TEG fabric, comprising 28-pair CNT thermoelectric array and a 0.55-mm-thick PVDF-HFP radiative-cooling membrane, achieves a *ΔT* of 37.1°C and an output voltage 47.9 mV under 1-sun illumination (Fig. 1f). Besides, the TEG fabric with 28-pair thermoelectric arrays can be connected to a voltage-boost circuit. Under standard 1-sun illumination, the system achieved an amplified output voltage of 2.0 V, demonstrating its capability to power various small wearable electronics including green light-emitting diodes and digital timers. Moreover, the power-generation performance of a larger 1 m × 0.2 m TEG fabric with a 560-pair thermoelectric array was tested between 10:00 and 16:00 under natural light. The output voltage peak is 6.67 V/m² at 12:30 PM and the peak power density is 0.20 mW/m² (Fig. 1g). Compared with previous TEG fibers, this TEG demonstrates superior performance and scalability, making it promising for future self-powered wearable applications.

By screen-printing CNT thermoelectric arrays with photothermal properties onto commercial white fabric and locally applying PVDF-HFP nanofiber radiative-cooling membranes, large-area radiation-modulated TEG fabrics with a significant *ΔT* were successfully fabricated. The synergistic combination of the CNT's photothermal performance and the PVDF-HFP's radiative-cooling properties enabled the TEG fabric to achieve a *ΔT* of 37 K and a high output voltage density of 6.67 V/m² under solar irradiation. However, the TEG fabric still faces limitations such as performance variability due to weather conditions and diurnal temperature fluctuations. While performance variability due to environmental conditions remains a limitation, this work establishes a practical framework for sustainable energy harvesting in wearable electronics. By synergizing photothermal and radiative-cooling properties, the fabric offers a scalable solution for powering low-energy devices, positioning it at the forefront of next-generation self-powered textiles for internet of things, healthcare monitoring and smart-clothing applications.
